# Thunderstorms and Lightning Accidents, etc.

**Published:** 1888-07-07

**Authors:** H. Newman Lawrence

**Affiliations:** Electrician to the Institute of Medical Electricity. 24, Regent Street, London, S.W.


### THUNDERSTORMS AND LIGHTNING ACCIDENTS.

As the season of thunderstorms and lightning accidents is now approaching, I hope you will kindly allow me to make known through your columns the fact that in the interest of science the Institute of Medical Electricity is very desirous of obtaining authentic information concerning lightning accidents, whether fatal or otherwise. I should therefore esteem it a favour if some of the many friends of humanity among your readers will assist us to investigate these phenomena by sending me such particulars of accidents of this nature as they may have personal or reliable knowledge of as soon after they occur as possible. Of course electrical and physiological details are what we most require, but reliable general information is often very valuable, and will be gratefully received.

H. Newman Lawrence, A.S.T.E. and E.,
Electrician to the Institute of Medical Electricity.
24, Regent Street, London, S.W.

